# Spectral Illusions

**Published:** 1831

**Authors:** 


### XXXIV.

### SPECTRAL ILLUSIONS.

THE following instance, related by a medical gentleman in whose person it occurred, is worthy of notice on more than one account. It appears that the gentleman in question spent some months of the last Summer (1830) in travelling over various parts of Britain, "undergoing much bodily exertion, often extending to a considerable degree of fatigue, and accompanied by its usual concomitant, a disinclination to mental exertion." During this period, he read little, which was contrary to his usual habits; and, in fact, using scarcely any intellectual exercise. On returning to Edinburgh, at the commencement of the Winter session, all active corporeal labour was discontinued—several hours daily were spent in dissection—and the remainder of the day in examining and arranging various plants and insects.

"While engaged in the latter occupation, it was of course necessary to examine minutely the composition and structure of the different objects, many of which were so small as to require the use of glasses. In short, I had made a sudden change from corporeal without intellectual exercise, to its opposite, or intellectual without corporeal; such exercise, too, being almost limited to the perceptive organs, as is evident from the manner of spending the day just mentioned. Shortly after this commenced, I began to feel, towards the evenings of several successive days, much heat and pain of eyes, with a disagreeable sensation of tightness and pain between the eyebrows, to such an extent as generally to prevent sleep, until after the repeated application of cold, by immersing my forehead in water. Notwithstanding the alleviation afforded by this, there still remained a tendency to start, awake suddenly, and apparently after having slept but a few minutes. On these occasions, it almost always appeared as if there were various strange objects in the room, uncouth and shapeless forms (if such an expression may be allowed), hardly referrible to any thing I could remember to have before seen. There were rarely more than two or three present at once, and they were commonly resolved into chairs or other real objects, after a minute or two of steady inspection. Occasionally, forms of a more distinct kind appeared; and once I had a lively representation of the white-robed figure to be seen in the Diorama of Holyrood Chapel, but it had ceased to be visible before it occurred to me where I had seen it before, or even that it was not entirely original. Just on first awaking, I never knew these appearances to be delusive, but a minute or two of time seemed to suffice for the gradual recalling and connecting of previous ideas with sufficient clearness to remind me that they *must* be so. Uutil I knew them to be illusions, the impression was rather disagreeable, and inclined to excite some feeling of apprehension. The knowledge of their ideal nature was almost always followed by their immediate disappearance or matamorphoses into chairs, curtains, moon-beams, or other realities. A few times this was not so immediate, and I was obliged once or twice to resort to the sense of touch in order to confirm the conclusions of reason. I believe that, at the moment of waking, there was a sensation as if I had received a blow on the forehead, but of this I was never sufficiently collected to be quite certain. These nocturnal startings from sleep, and illusive appearances, lasted about ten days; after which, having finished the examination and arrangement of my botanical and natural-historical preparations, and commencing to attend the College lectures, a more varied excitement of the intellectual powers, and somewhat less sedentary habits, supervened, and the restless sleep and illusions gradually ceased, and were soon almost forgotten; but the cessation was so gradual that I should be quite unable to say when it was completed."*

About two months afterwards he had again occasion to dévote himself almost entirely, for a short time, to intellectual pursuits; and again the fanciful visions of the night returned. Our readers will readily draw their own conclusions from the foregoing narrative.

---

* Phrenological Journal, No. XXVIII, June 1, 1831.

